# A cross-sectional survey of knowledge, attitude, and willingness to engage in spontaneous reporting of adverse drug reactions by Korean consumers

**DOI:** 10.1186/s12889-020-09635-z

**Published:** 2020-10-08

**Authors:** Seungyeon Kim, Yun Mi Yu, Myoungsoon You, Kyeong Hye Jeong, Euni Lee

**Affiliations:** 1 College of Pharmacy & Research Institute of Pharmaceutical Sciences, Seoul National University, Seoul, 08826 Republic of Korea; 2 Department of Pharmacy and Yonsei Institute of Pharmaceutical Sciences, College of Pharmacy, Yonsei University, Incheon, 21983 Republic of Korea; 3 Department of Pharmaceutical Medicine and Regulatory Sciences, Colleges of Medicine and Pharmacy, Yonsei University, Incheon, 21983 Republic of Korea; 4 Department of Public Health Sciences, Graduate School of Public Health, Seoul National University, Seoul, 08826 Republic of Korea; 5 College of Pharmacy, Chung-Ang University, Seoul, 06974 Republic of Korea

**Keywords:** Pharmacovigilance, Adverse drug reaction, Consumer, Spontaneous reporting, Attitude, Awareness, Self-efficacy

## Abstract

**Background:**

Spontaneous reporting (SR) of adverse drug reactions (ADRs) from patients can be considered as a valuable activity providing both objective and subjective data. However, improving the rate of under-reporting has been a major challenge to ensure successful operation of the SR system. This study aimed to assess knowledge, attitude, and intent to report ADRs and explore the factors contributing to consumers’ reporting intent in South Korea.

**Methods:**

Self-administered questionnaire was collected from a sex-, age-, and regionally stratified nationwide convenience sample of consumers using a commercial panel in December 2018. Univariate and multivariate logistic regression analyses were used to explore the factors contributing to the intent to report ADRs by consumers.

**Results:**

A total of 1000 respondents were enrolled in the survey; 50.9% were males and the mean age was 44.4 (standard deviation, 13.3) years. While less than 15% of the respondents were aware of the SR system and even fewer (3.4%) had actual experience of SR, however, 59.2% expressed their intent to report ADRs. The positive attitude (adjusted odds ratio [aOR] 3.972, *p* < 0.001), awareness of the SR system (aOR 2.102, *p* < 0.01), self-efficacy for SR (aOR 1.956, *p* < 0.001), and experiences related to ADR counselling with healthcare professionals (OR 2.318, *p* < 0.001) are the significant factors contributing to reporting intent.

**Conclusions:**

Findings of this study highlight the need for increasing the awareness of the SR system among consumers and empowering them to report ADRs by themselves, which would ultimately improve the drug-safety environment.

## Background

Spontaneous reporting (SR) of adverse drug reactions (ADRs) is one of the fundamental activities by which pharmacovigilance (PV) strives to ensure the safe use of medications and detects safety signals. Direct voluntary reporting by consumers has the advantage of increasing the overall reporting rates and obtaining detailed information [1] as reports from patients could serve as a valuable source of both objective data and subjective real-life experience from their perspectives. According to some research from the UK and Netherlands, consumer reports provided comparable seriousness and associations in comparison to reports from healthcare professionals (HCPs) [2–4]. Although some HCPs and researchers can be doubtful about the accuracy and the quality of ADR reports from the consumers, the contribution of direct consumer reporting to pharmacovigilance as a signal detecting tool cannot be ignored [1]. For these reasons, recent literature has continuously recognised the importance of consumer/patient reporting, and many countries are encouraging patients/consumers to report ADRs through their national reporting systems, such as the Yellow Card Scheme in the United Kingdom (UK) and the Food and Drug Administration Adverse Event Reporting System in the United States (US) [5].

Since the launch of the SR system in South Korea in 1988, the government has made systematic efforts to structure a PV system with active surveillance, such as, the establishment of the Korea Institute of Drug Safety and Risk Management (KIDS) [6]. KIDS has strategically expanded the number of regional drug safety centres from three to 27 since the baseline year, 2006 [7]. The latest effort from the Korean government has been the implementation of the relief system in 2014 for the victims of ADRs [8]. Although the reporting rate of ADRs in Korea has increased, with 1,599,212 accrued reports from 1989 to 2018, reports from consumers comprised only about 8% of the total reports [9], a rate which was considerably lower than that of the US (45%), Canada (30%), UK (20%), and Netherlands (55%) [10, 11].

Improving the rate of under-reporting has been a major challenge for the successful functioning of the SR system globally [12]. Therefore, for several decades, a number of published studies have tried identifying the factors contributing to the reporting behaviour by HCPs and consumers [13–15]. Some recent studies that have analysed consumers’ reporting behaviour [16–18] have identified distinctive barriers and motives for ADR reporting, such as poor awareness of the SR system and altruism. The majority of the published studies on PV activities by consumers have largely been limited to descriptive analyses based on a small number of participants, and have seldom been driven by a conceptual framework that allows for the comprehensive evaluation of factors contributing to the reporting behaviour [16, 19]. Therefore, we conducted this study to assess the knowledge, attitude, and intent in relation to the voluntary reporting of ADRs, and explore the factors that contribute to the consumers’ reporting intention in South Korea based on a conceptual model.

## Methods

### Study design and study population

A cross-sectional survey was conducted by a web-based recruitment method using a consumer panel with a nationwide convenience sample. A stratified sampling scheme was used to reflect the sex, age, and region corresponding to the population distribution in South Korea [20]. The consumer panel operated by the Panel Marketing Research Interactive Company was employed to recruit the participants. This was the largest panel, including 1.5 million people in South Korea, and was considered to provide participants who are highly representative of the population by utilising a systematic management system that includes regular pre-survey and double-check system [21]. A more detailed description of the panel has been published elsewhere [22]. Further, a self-administered questionnaire was accessible on the online website and the participants could start the survey by clicking the button by themselves. The survey questionnaire comprising a total of 57 questions was divided into 2 screening questions and 55 main questions. The eligible participants should not be healthcare professionals and had to be adults who were 19 years and older as filtered by screening questions. The data was collected in December 2018.

All survey participants provided their informed consent prior to participation in the study. The study protocol was approved by the Institutional Review Board (IRB) of Seoul National University (IRB No. E1811/003–013). Following the editorial policies of the BioMed Central, the study was conducted according to Strengthening the Reporting of Observational studies in Epidemiology guidelines and the checklist was provided (Supplementary Table 1).

### Questionnaire development

The questionnaire developed for this study used the mixed theoretical model [23], which combines the knowledge-attitude-practices model [24] and the theory of satisfaction of needs [25], was applied in developing the questionnaire. Since this model was designed to explain both, the intrinsic and extrinsic conditions [23], the questions relating to personal information, clinical background, knowledge, and attitude toward SR were treated as constituting intrinsic conditions, whereas questions relating to the relationships with their family members, HCPs, and healthcare administration were treated as constituting extrinsic conditions. The personal information collected includes sex, age, residential area, level of education, and monthly income, and information on clinical background comprised questions pertaining to the history of medication, family members of HCPs, experiences related to ADRs. The experience related to ADRs consisted of the five questions: (1) ‘concerns about the occurrence of ADRs’, (2) ‘seeking information on ADRs’, (3) ‘reading insert paper’, (4) ‘participants’ own experience of ADRs or that of family members’, and (5) ‘requests to HCPs for counselling in drug safety’.

As studies showed that consumers needed more steps to identify their symptoms as ADRs than HCPs [19], we incorporated the knowledge or ability to recognise ADRs into our conceptual framework reflecting consumers’ perspectives. Based on the framework, five questions were formulated, addressing the objective knowledge of ADRs [19, 26]. For the case of knowledge about SR, the questions about their awareness of the SR system and the relief scheme supported by the Korean government were included and the questions that measure self-efficacy, indicating perceived ability to spontaneously report, were considered. During the survey, a reporting form was given to each participant to find out whether he/she could complete SR by filling out the forms and how easy was it to report ADRs using the reporting form.

The questions pertaining attitude to SR addressed participants’ motives and the barriers participants face while reporting ADRs. These questions were guided by published studies assessing participants’ intent and behaviour to go through the SR process [16, 27, 28]. The questions addressing the motives to report ADRs included questions on the necessity for reporting ADRs, duty to report ADRs, and expectations of a positive impact from reporting ADRs. The barriers to consumers’ ADR reporting were assessed based on responses to the following: (1) ‘cannot recognise the ADR’, (2) ‘no serious ADRs’, (3) ‘ADRs resolved’, (4) ‘no personal benefit’, (5) ‘no real improvement in the system’, (6) ‘not my job’, (7) ‘counsel with HCPs instead of SR’, and (8) ‘breach of privacy’.

The questions relating to ADR knowledge, self-efficacy, and attitude to SR were measured using a 5-point Likert-type scale, with 5 points allocated to ‘strongly agree’, 4 points to ‘agree’, 3 points to ‘neutral’, 2 points to ‘disagree’, and 1 point to ‘strongly disagree’. We measured the frequency of SR experiences and collected the participants’ intent to report ADRs, which could be proportional to the actual reporting behaviour. Further, the face validation was completed by five pharmacists, five members of the public, and four researchers involved in this study to test the appropriateness of the questions and improve their clarity. Lastly, an additional pilot testing including 30 consumers was completed to enhance the clarity of the survey questions further. Data for the pilot study was not included in the analysis of the main results.

### Sample size

Based on the survey results [29] that 14.5–34.7% of the participants had experienced ADRs and that about 59% of the participants reported patients and consumers as suitable reporters for ADRs, the target sample size of 1000 respondents was calculated as being adequate to detect a 10% difference in reporting intent (with 80% power and 5% α-error). The sample size calculation was performed using Epi Info™ 7.1.5 (Centers for Prevention and Control, Atlanta, GA).

### Statistical analysis

To assess the internal consistency reliability and construct validity of the questionnaire on experiences related to ADRs, knowledge of ADRs, and attitude toward SR, the Cronbach’s α coefficient and exploratory factor analysis were used. The Cronbach’s α coefficient ≥ 0.7 is an acceptable cut-off for internal consistency and the data is considered fit for factor analysis if the Kaiser-Meyer-Olkin measure of the sampling adequacy is over 0.7 and a *p*-value in < 0.001 in Bartlett’s Test of Sphericity. The numbers of factors for the analysis were determined based on eigenvalues above 1 being the reference and the scree plot. Items that indicated a loading ≥0.4 were considered as the corresponding factors [30, 31].

Descriptive statistics were used to summarise the respondents’ characteristics. The univariate and multivariate logistic regression analyses were performed to explore the predictive factors for reporting intent. Further, the degree of association between reporting intent and the factors was presented as odds ratios (ORs) with corresponding 95% confidence intervals (CIs). All statistical analyses were performed using SAS version 9.4 (SAS Institute Inc., Cary, N.C., USA) and the level of statistical significance was set at *p* < 0.05.

## Results

### Respondents’ characteristics

A total of 1000 respondents were enrolled in the study, and the questionnaire distribution was closed as the target sample size was attained. Almost 51% of the respondents were male and the mean (± standard deviation) age was 44.4 (± 13.3) years. Almost half of the respondents (45.0%) lived in metropolitan areas and the majority (74.5%) were graduates from the university.

With regard to ADRs-related experiences, nearly 65% of the respondents felt concerned about the occurrences of ADRs, and less than half of them (43.2%) read insert paper. Over half of the respondents (53.6%) reported of either having experienced ADRs themselves or experienced by family members. However, only 34 respondents (3.4%) reported actual experience of SR and 592 (59.2%) expressed their intent to report ADRs. Details of respondents’ characteristics are shown in Table 1.

**Table 1:** Demographic characteristics and clinical background of survey respondents (*n* = 1000)

Characteristics	Number (%)
Sex	
Male	509 (50.9)
Female	491 (49.1)
Age	
mean ± SD^a^	44.4 ± 13.3
Under 40 years	380 (38.0)
40–65 years	532 (53.2)
Over 65 years	88 (8.8)
Residential area	
Metropolitan	450 (45.0)
Rural	550 (55.0)
Level of education	
Middle school or below	28 (2.8)
High school graduate	227 (22.7)
College or above	745 (74.5)
Average monthly income^b^	
1st quintile (highest)	77 (7.7)
2nd quintile	199 (19.9)
3rd quintile	258 (25.8)
4th quintile	223 (22.3)
5th quintile (lowest)	243 (24.3)
Continuous medication over 3 months	795 (79.5)
Ever used one or more medications within the recent year	866 (86.6)
Having healthcare professional family members or relatives	258 (25.8)
Experiences related to ADRs	
Experience of concerns about the occurrence of ADRs	642 (64.2)
Experience of seeking drug safety information	581 (58.1)
Experience of reading insert paper thoroughly	432 (43.2)
Experience with ADRs	536 (53.6)
Experience of request to healthcare professionals for counselling in drug safety	839 (83.9)
Experience of spontaneous adverse drug reaction reporting	34 (3.4)
Intent to report adverse drug reaction	592 (59.2)

^a^The mean age (± SD) is presented as a numeric value in the years unit

^b^The income data are based on national-level statistics provided by KOSIS^20^

*ADRs* Adverse drug reactions, *SD* Standard deviation

### Knowledge of and attitude towards ADR reporting

Overall, 47.4% of the respondents correctly answered all the questions related to knowledge of ADRs. About 14 and 10.4% of the respondents were aware of the SR system and relief scheme for ADR, respectively. In terms of self-efficacy of SR, 43.5% of the respondents agreed to the fact that reporting ADR as they use the reporting form was easy and about 56% claimed that they were able to fill out the forms to complete their ADR reporting.

Most of the respondents (93.9%) agreed on the necessity of the SR system and 72.8% agreed on the general public’s duty to report ADRs. Almost two-thirds of respondents anticipated that reporting ADRs would prevent similar ADRs (77.6%), improve drug safety (75.8%), and improve healthcare service (64.5%). Approximately two-thirds of the respondents agreed to the fact that barriers related to the detection of the ADRs, including ‘cannot recognise the ADRs’ (75.6%), ‘no serious ADRs’ (67.0%), and ‘ADRs resolved’ (63.5%) appeared to be hindering factors for reporting the ADRs. However, fewer respondents agreed to the fact that personal thoughts on the SR system such as ‘breach of privacy’ (35.6%), ‘not my job’ (41.6%), and ‘no personal benefit’ (54.3%) were barriers to report ADR (Supplementary Table 2).

### Reliability and validity

Cronbach’s α was obtained from questions pertaining to experiences related to ADRs (0.62), knowledge of ADRs (0.70), motives of SR (0.72), and barriers against SR (0.76). Except for the alpha value of questions about experiences related to ADRs, other alpha values showed satisfactory internal consistency. These questions were confirmed to be suitable for exploratory factor analysis as there was an exceeding value of 0.7 in the Kaiser-Meyer-Olkin measure and the Bartlett’s Test of Sphericity (*p*-value < 0.001) was statistically significant. The factor loading for each item is provided in Table 2. The factor analysis indicated that the items of ADRs-related experiences and knowledge loaded on a single factor, explaining a total variance of 40.49 and 45.84%, respectively. The items from motives and barriers on SR were loaded on two factors, explaining a cumulative variance of 69.84 and 54.16%, respectively. The two factors of motives for reporting were categorised as ‘expectation from the SR system’ and ‘a sense of necessity and duty to report’. Further, the two factors of barriers on SR can be defined as ‘obstacles related to the detection of ADRs’ and ‘obstacles resulting from personal thoughts on the SR system’.

**Table 2:** Internal consistency reliability and factor loading on the items for experience and knowledge of adverse drug reaction and attitude on spontaneous reporting (*n* = 1000)

Item	Total correlation coefficient	Factor	
		1	2
**Experiences related to ADRs (0.62)**^**a**^			
Experience of concerns about the occurrence of ADRs	0.45	0.73	
Experience of seeking drug safety information	0.51	0.77	
Experience of reading insert paper thoroughly	0.27	0.50	
Experience with ADRs	0.37	0.63	
Experience of requests HCPs for counselling in drug safety	0.28	0.50	
Eigenvalue		2.02	
Cumulative variance explained (%)		40.49	
**Knowledge of ADRs (0.70)**^**a**^			
All medications can cause adverse effects	0.47	0.68	
Adverse effects can occur even if a medication is administered correctly	0.40	0.61	
Adverse effects can occur when a medication is double-dosed	0.34	0.54	
Adverse effects can occur when a medication is taken more frequently	0.57	0.79	
Adverse effects can occur when medications are stopped abruptly	0.51	0.74	
Eigenvalue		2.29	
Cumulative variance explained (%)		45.84	
**Attitude on motives of SR (0.72)**^**a**^			
Necessity of reporting ADRs	0.37	0.19	0.78^b^
Duty to report ADRs	0.31	0.08	0.83^b^
Expectation of preventing similar ADRs from occurring in others	0.57	0.84^b^	0.13
Expectation of improving drug safety	0.54	0.81^b^	0.12
Expectation of improving healthcare service	0.62	0.86^b^	0.16
Eigenvalue		2.42	1.07
Cumulative variance explained (%)		48.40	69.84
**Attitude on barriers against SR (0.76)**^**a**^			
Cannot recognise ADRs	0.43	0.21	0.66^b^
No serious ADRs	0.45	0.09	0.83^b^
ADRs resolved	0.43	0.10	0.78^b^
No personal benefit	0.53	0.68^b^	0.26
No real improvement in system	0.55	0.71^b^	0.26
Not my job	0.45	0.73^b^	0.06
Counsel with HCPs instead of SR	0.52	0.56^b^	0.37
Breach of privacy	0.34	0.67^b^	−0.05
Eigenvalue		3.05	1.29
Cumulative variance explained (%)		38.07	54.16

^a^Cronbach’s alpha values are presented

^b^Item loading > 0.4

*ADRs* Adverse drug reactions, *HCPs* Healthcare professionals, *SR* Spontaneous reporting

### Contributing factors for reporting intent

Contributing factors for the intent to spontaneously report were evaluated and presented in Table 3. Male respondents (adjusted OR [aOR], 1.477; 95% CI, 1.104–1.976) and respondents with high monthly income were more likely to express their intent for SR (aOR, 1.427; 95% CI, 1.074–1.896). The respondents’ experience with ADRs was significantly associated with the intent to report ADRs (aOR, 1.778; 95% CI, 1.327–2.381). In the crude analysis, respondents who were aware of the SR system and the relief scheme were more likely to carry the intent to report ADRs. However, after other variables were adjusted, only awareness of the relief scheme remained as a statistically significant factor (aOR, 2.102; 95% CI, 1.233–3.584). The respondents who believed that they had the ability to spontaneously report ADRs by agreeing with both the self-efficacy measuring items were more likely to show their intent for SR than those who agreed on just one item or less (aOR, 1.956; 95% CI, 1.467–2.609).

**Table 3:** Contributing factors for intent to spontaneously report on adverse drug reactions (n = 1000)

Characteristics	Intent to spontaneously report ADRs, n (%)		Crude OR (95% CI)	aOR (95% CI)^a^
	No (***n*** = 408)	Yes (***n*** = 592)		
***Demographic and clinical characteristics***				
Sex				
Male	200	309	1.136 (0.882–1.462)	1.477 (1.104–1.976)^**^
Female	208	283	1.0	**1.0**
Age group				
Under 40 years	157	223	1.0	1.0
40–65 years	212	320	1.063 (0.813–1.389)	1.065 (0.774–1.466)
Over 65 years	39	49	0.885 (0.554–1.412)	1.012 (0.582–1.760)
Residential area, n (%)				
Metropolitan area	184	266	0.993 (0.771–1.28)	0.938 (0.703–1.251)
Rural area	224	326	1.0	1.0
Level of education				
Low (less than high school graduate)	132	123	**1.0**	1.0
High (more than university attendee)	276	469	1.824 (1.368–2.430)^***^	1.376 (0.980–1.932)
Average monthly income^b^				
Low	220	246	**1.0**	**1.0**
High	188	346	1.646 (1.276–2.122)^***^	1.427 (1.074–1.896)^*^
Continuous medication				
No	177	238	1.0	1.0
Yes	231	354	1.330 (0.976–1.812)	0.947 (0.651–1.378)
Recent medication within one year				
No	71	63	**1.0**	1.0
Yes	337	529	1.769 (1.227–2.550)^**^	1.439 (0.927–2.235)
Have HCPs as family members or relatives				
No	389	558	1.0	1.0
Yes	19	34	1.280 (0.956–1.715)	0.899 (0.641–1.262)
Number of experiences related to ADRs				
≤ 3 items	276	291	**1.0**	**1.0**
4–5 items	132	301	2.163 (1.663–2.812)^***^	1.778 (1.327–2.381)^***^
***Knowledge and attitude variables***				
Amount of accurate knowledge on ADRs				
≤ 4 items	244	282	**1.0**	1.0
5 items	164	310	1.636 (1.267–2.111)^***^	1.304 (0.968–1.755)
Awareness of SR system				
No	372	484	**1.0**	1.0
Yes	36	108	2.306 (1.545–3.442)^***^	1.348 (0.811–2.240)
Awareness of relief scheme for ADRs				
No	386	510	**1.0**	**1.0**
Yes	22	82	2.821 (1.73–4.599)^***^	2.102 (1.233–3.584)^**^
Self-efficacy of SR				
0–1 item	247	231	**1.0**	**1.0**
2 items	161	361	2.397 (1.852–3.104)^***^	1.956 (1.467–2.609)^***^
Number of positive attitude items on expectations of SR				
≤ 2 items	231	185	**1.0**	**1.0**
3 items	177	407	2.871 (2.210–3.730)^***^	2.027 (1.521–2.703)^***^
Positive attitude on necessity and duty for SR				
No	188	92	**1.0**	**1.0**
Yes	220	500	4.644 (3.456–6.240)^***^	3.972 (2.896–5.449)^***^
Concerns on the barriers related to the detection of ADRs				
≤ 2 items	181	318	0.687 (0.533–0.885)^**^	0.978 (0.720–1.330)
3 items	227	274	**1.0**	1.0
Concerns on the barriers derived from personal thoughts on the SR system				
≤ 3 items	182	291	0.833(0.647–1.073)	1.076 (0.797–1.454)
4–5 items	226	301	1.0	1.0

^a^Multivariate analysis adjusted for all variables listed in the table

^b^1^st^ quintile (highest) to 3rd quintile of the average monthly income were categorised as a high-income group

^*^p < 0.05, ^**^p < 0.01, ^***^p < 0.001

*ADRs* Adverse drug reactions, *CI* Confidence interval, *HCPs* Healthcare professionals, *aOR* Adjusted odds ratio, *SR* Spontaneous reporting

The positive attitudes toward motives, including expectations, necessity, and duty of SR, were associated with the intent to report ADRs. The associations for items related to recent medication, accurate knowledge of ADRs, and concerns with barriers to the detection of ADRs were not apparent in the multivariate logistic regression model.

**Fig. 1:**
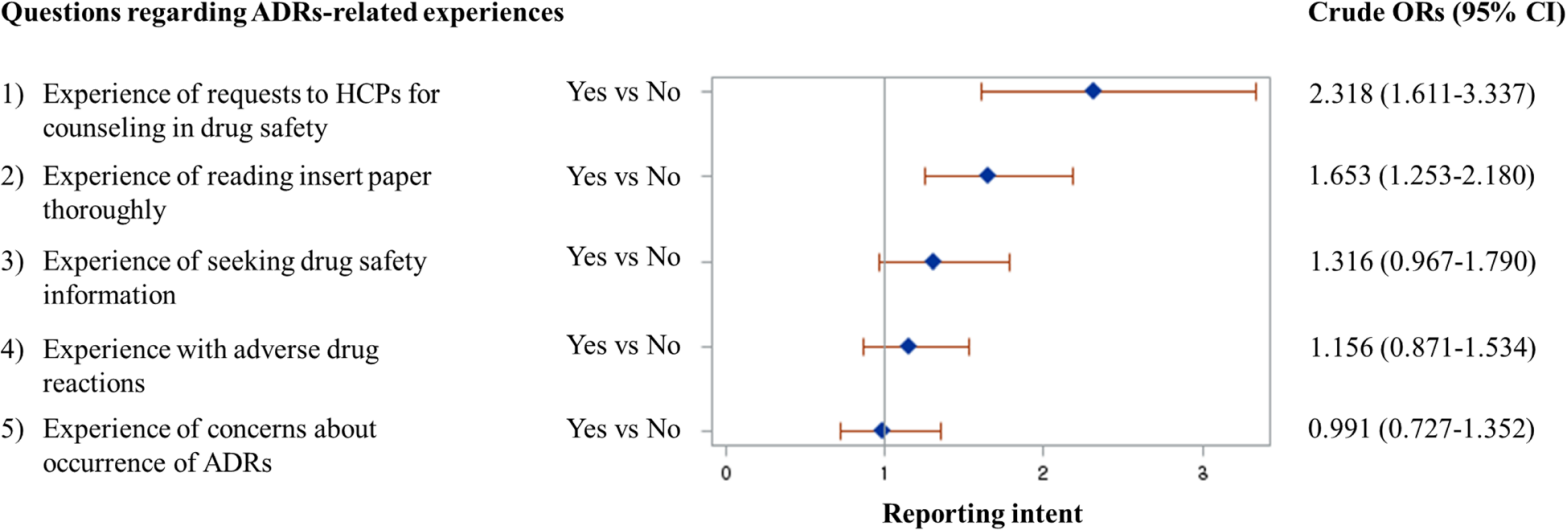
The associations between items regarding experience related to ADRs and the intent to report ADRs. ADRs: adverse drug reactions

The association between each specific type of ADRs-related experiences and the intent for SR has been evaluated and presented in Fig. 1. Of the five different types of ADRs-related experiences, ‘requests to HCPs for counselling in drug safety’ (OR, 2.318; 95% CI, 1.611–3.337) and ‘reading insert paper’ (OR, 1.653; 95% CI, 1.253–2.180) were found to be the significant predictive factors for expressing intent to report ADRs.

## Discussion

To the best of our knowledge, this is the first nationwide survey to explore the factors contributing to the intent to report ADRs by consumers in South Korea. Although many studies have focused on reporting HCPs’ perspectives on voluntary ADR reporting [13–15], we believe that a relatively small number of studies provided an understanding of the consumers’ perspectives. Therefore, a study like this quantitative survey could add valuable insights on consumers’ attitude and intents toward spontaneously reporting ADRs. An additional strength of this study is that the survey was developed using a conceptual framework, which embodies the potential determinants affecting the intent to engage in SR behaviour [32]. Thus, we believe that the findings from our study could not only provide more reliable information to the existing body of knowledge [16, 18, 33] but also a theoretical basis for future studies.

Our study demonstrated that enhancing awareness about the SR system is a strong predictor of reporting intents. Literature shows that multilateral efforts have been made in enhancing the awareness and supporting consumer/patient outreach for ADR reporting behaviours in various countries [34, 35]. According to a Dutch study [34], media attention toward the benefits and risks of specific drugs led to increased reporting of patient ADRs. Also, some studies reported the media to have an influence on patient behaviour [36, 37]. Recently, as the paradigm of communication approaches in public relations has shifted from traditional media to new forms of communication methods [38], government-level outreaching initiatives that use various social media (e.g., Facebook and Instagram) were observed in South Korea and other countries [39, 40]. Both, traditional methods and other innovative communication channels could improve awareness about the SR system and educate consumers on the benefits of ADR reporting.

Our findings on the relationship between policy implementation and consumers’ behavioural intent are also noteworthy. Consumers’ knowledge of an ADRs-related policy item, such as the public relief scheme implemented in 2014, was a significant contributing factor for reporting intent. Although Japan, Taiwan, New Zealand, and Nordic countries as well as South Korea have implemented government-level compensation programmes [8], no study has documented the predictive relationship between the programmes and consumers’ reporting behaviour or willingness to report. These results may be attributed to the compensation concept of the relief scheme, and the findings were consistent with literature reporting the lack of personal financial gains as a barrier for SR [16, 28]. Therefore, public relations highlighting personal and tangible gains from the consumers’ perspectives may attract public attention and perhaps can be a potential solution for increasing the intent to report and actual reporting behaviour.

Empowering consumers to attain the self-efficacy of SR can be essential to improve consumer engagement in pharmacovigilance. Despite a user-centred interface, with a guided dropdown menu in the online reporting system offered in South Korea, about half of the respondents in our survey stated that filling out the reporting form was not easy. It could potentially be because symptom options under the dropdown menu were given in technical terms. A published study on medication literacy in South Korea [41] reported that the proportion of adults who did not understand symptom-related vocabulary was higher in South Korea than in other countries such as US, Canada, Netherlands and Germany. Therefore, as a study evaluating the usability of the UK Yellow Card Scheme [42], we believe that a comprehensive assessment of the current reporting system in South Korea is needed from the users’ perspectives, especially targeting the consumers with low self-efficacy. Also, a stepwise modification of the reporting system should be considered by adopting easier terminology from the patients’ perspective [43].

The patient-reported outcomes (PROs) of adverse events (AEs), i.e., PRO-AE data, have become increasingly important in clinical practice, especially for those symptomatic AEs often undetected by HCPs [43, 44]. However, patients feared that their complaints of potential ADRs would be either neglected, underestimated, or denied by HCPs [45–47]. While these negative experiences with HCPs can be a barrier to SR, our study demonstrated the critical role of HCPs in drug safety-related counselling, predictive of reporting intent. Unrecognised ADRs could be brought to HCPs’ attention and patients’ awareness of specific ADRs could be raised [16, 48] by proactive communication between HCPs and patients. Therefore, we believe that HCPs can be key people in contributing to the accumulation of real-world safety data to enhance the quality of patient-centred care.

This study has several limitations. First, our study was based on a convenience sample, wherein the respondents were recruited online using a commercial panel. Thus, there is a possibility for people not familiar with the internet being underrepresented. Although we tried to minimise a potential selection bias by stratifying the sample to reflect the distribution of the South Korean population, limited generalisability cannot be ignored. Second, the conceptual model adopted in our study is based on the mixed theoretical model for HCPs because the consumer-specific conceptual frameworks of ADR reporting were scarce. Therefore, we tried to incorporate factors focusing on consumer behaviours into the survey questions, as suggested in the literature [16]. Third, while the Cronbach’s α for items of experiences related to ADRs was below the acceptable value of 0.7, the values of Cronbach’s α for other items were above the cut-off. The dichotomous responses to these items might be a reason for the low Cronbach’s α [15]. Fourth, this study did not consider the effect of response biases prevalent in self-report survey research. Since our study was carried out as an online survey, respondents can search the correct answers to questions on knowledge about ADR and SR. However, the possibility of the biases influencing our results would not be considerable as the respondents lacked incentives or financial gains to provide the correct answer and would not have sufficient time to find the correct answer as indicated by an estimated average response time of 6 s per question from our in-house survey summary report. Fifth, the inability to derive causal inference is the inherent limitation of the cross-sectional study design. Finally, our study could not evaluate the predictors of actual ADR reporting because only a few respondents had the experience. Therefore, we believe that further prospective and longitudinal studies are needed to determine the predictive factors that connect reporting intent to actual reporting behaviour.

## Conclusions

Our survey on consumers found less than 15% of the respondents to be aware of the SR system, and even fewer respondents to have direct reporting experience. However, more than 90% of the respondents agreed on the necessity of SR, and over half of the respondents showed their intent to report ADRs in the future. As the self-efficacy of SR and experience related to ADR counselling with HCPs were significantly associated with reporting intent, the roles of HCPs should be emphasized. Empowering consumers/patients and guiding them in the reporting process can improve the under-reporting of ADRs by consumers, and ultimately enhance the drug-safety environment in South Korea.

## Supplementary information

**Additional file 1: Supplementary Table 1.** STROBE checklist of items reporting cross-sectional studies.

**Additional file 2: Supplementary Table 2.** Proportions of the survey responses on attitude towards motives and barriers.

## Data Availability

The datasets used and/or analysed during the current study are available from the corresponding author on reasonable request.

## Abbreviations

ADRs: Adverse drug reactions
AEs: Adverse events
CIs: Confidence intervals
HCPs: Healthcare professionals
IRB: Institutional Review Board
KIDS: Korea Institute of Drug Safety and Risk Management
ORs: Odds ratios
PROs: Patient-reported outcomes
PV: Pharmacovigilance
SR: Spontaneous reporting
UK: United Kingdom
US: United States
