# Supplementary material for: A cross-sectional survey of knowledge, attitude, and willingness to engage in spontaneous reporting of adverse drug reactions by Korean consumers
Source: BMC Public Health. 2020 Oct 8;20:1527. doi: 10.1186/s12889-020-09635-z (PMC7545860; doi:10.1186/s12889-020-09635-z)
Supplement: Supplementary file 2 — Additional file 2: Supplementary Table 2. Proportions of the survey responses on attitude towards motives and barriers. [file 12889_2020_9635_MOESM2_ESM.docx]

Supplementary Table 2. Proportions of the survey responses on attitude towards motives and barriers^a^

| **Questions** | **Response (%)** | | | | |
| --- | --- | --- | --- | --- | --- |
|  | **Strongly agree** | **Agree** | **Neutral** | **Disagree** | **Strongly disagree** |
| ***Attitude on motives against SR***^b^ | | | | | |
| Expectation of preventing similar ADRs from occurring in others | 15.5 | 62.1 | 18.4 | 3.5 | 0.5 |
| Expectation of improving drug safety | 14.5 | 50 | 26.8 | 8.1 | 0.6 |
| Expectation of improving healthcare service | 23.9 | 51.9 | 19.3 | 4.3 | 0.6 |
| ***Attitude on barriers of SR*** | | | | | |
| Cannot recognise ADRs | 14.3 | 61.3 | 20.4 | 3.7 | 0.3 |
| No serious ADRs | 10.9 | 56.1 | 24.6 | 8.1 | 0.3 |
| ADRs resolved | 7.6 | 55.9 | 29.4 | 6.7 | 0.4 |
| No personal benefit | 11.8 | 42.5 | 33.1 | 11.7 | 0.9 |
| No real improvement in system | 16.9 | 41.2 | 30.5 | 10.6 | 0.8 |
| Not my job | 7.9 | 33.7 | 37.6 | 18.9 | 1.9 |
| Counsel with HCPs instead of SR | 11.3 | 51.3 | 30 | 6.6 | 0.8 |
| Breach of privacy | 6.8 | 28.8 | 41.5 | 19.9 | 3 |

^a^The responses were collected in a 5-point Likert scale.

^b^As the questions related to the necessity and duty of reporting were measured using dichotomous values, they were not presented in this table.

ADRs: adverse drug reactions; HCPs: healthcare professionals; SR: spontaneous reporting.
